# Probing Heterogeneous Catalytic Reactions via Tip-Enhanced Raman Spectroscopy: Recent Progress and Future Perspectives

**DOI:** 10.1021/cbmi.5c00065

**Published:** 2025-11-17

**Authors:** Yusheng Zhang, Yuqin Xu, Jing-Juan Xu, Li-Qing Zheng

**Affiliations:** State Key Laboratory of Analytical Chemistry for Life Science, School of Chemistry and Chemical Engineering, 12581 Nanjing University, Nanjing 210023, People’s Republic of China

**Keywords:** heterogeneous catalysis, tip-enhanced Raman spectroscopy, active sites, chemical selectivity, operando studies, activation pathway, electrocatalysis, spatial resolution

## Abstract

Heterogeneous catalysis is pivotal to modern chemical industries, and molecular-level insights into catalytic processes are essential for developing highly efficient catalysts and advancing energy conversion technologies. Tip-enhanced Raman spectroscopy (TERS), which integrates scanning probe microscopy with plasmon-enhanced Raman spectroscopy, provides chemical and topographic information simultaneously with exceptional sensitivity and nanoscale spatial resolution. This technique is ideally suited for the nanoscale chemical characterization of solid catalysts, enabling direct structure–performance correlations. In this review, we first introduce the fundamental principles of TERS, and then highlight its key applications in probing heterogeneous catalysis, focusing on critical aspects such as active sites, molecular activation pathways, conversion efficiency, chemical selectivity, and operando studies. We conclude by discussing current challenges and potential strategies to advance TERS in heterogeneous catalysis, and by outlining future directions for the field.

## Graphical abstract

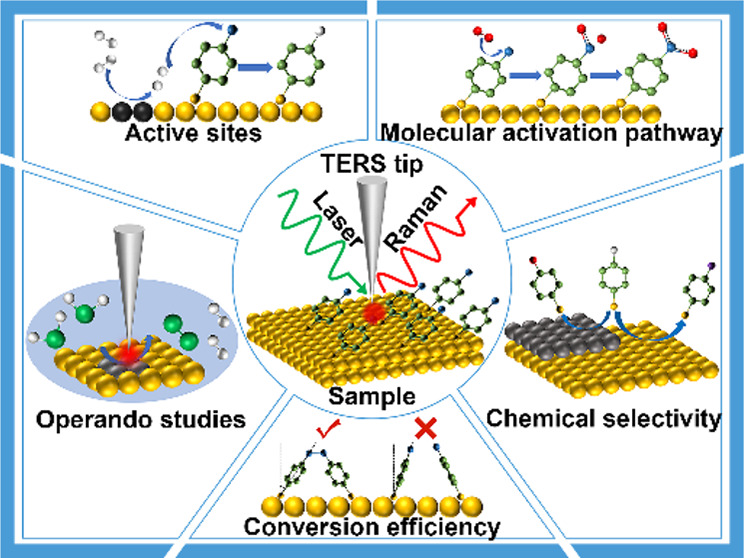

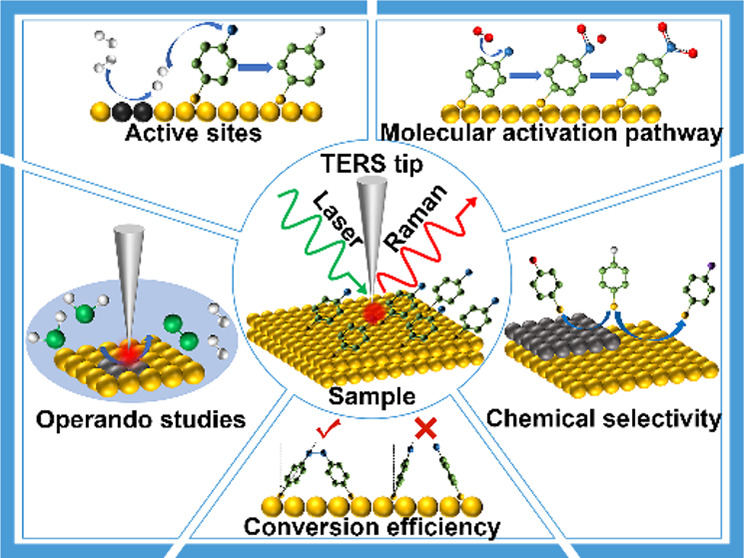

## 1 Introduction

Heterogeneous catalysis lies at the core of the modern chemical industry and has driven many technological advancements with the aim of making a more sustainable society.^1–3^ To date, heterogeneous catalysis has been widely used in numerous fields, including petrochemical refinement, pharmaceutical manufacture, environmental protection, and fine chemical synthesis.^4–6^ In heterogeneous catalysis, solid catalysts such as supported or unsupported metal catalysts,^7–10^ zeolites,^11,12^ and transition metal dichalcogenides (TMDCs),^13^ are extensively used and play key roles in achieving high catalytic activity and selectivity. Solid catalysts react with molecules in the liquid or gas phase during catalytic processes. Various highly dynamic processes in time and space may occur at the interface between the surface of the catalysts and the reactants, including submonolayer molecular adsorption, diffusion, electron transfer (generation of intermediates), and desorption of products on the catalyst surface.^14^ In the above processes, all the molecular species near the catalysts’ surface form the complex interfacial region. Understanding the physicochemical properties of these interfacial processes is crucial in controlling the catalytic activity and selectivity. The morphology and electronic structure of the solid catalysts inherently determine their catalytic performance. For example, local structures, including step edges, corners, kinks, adatoms, and defects, are often considered highly active sites where charge transfer between the molecules and substrates usually occurs.^15,16^ In addition, surface reconstruction and relaxation of solid catalysts may occur during catalysis. Therefore, it is necessary to establish the relationship between the topographic and electronic structure of solid catalysts and their chemical performance at the molecular to atomic level, which allows for the improvement of the rational design of highly efficient and selective catalysts.^17–20^

A complete understanding of the interfacial processes during heterogeneous catalysis calls for techniques that can provide information about the surface with a molecular fingerprint and nanometer-scale spatial resolution. With the development of surface science, various analytical tools have been employed to characterize the surface structure and properties of solid catalysts. Techniques include *in situ* electron microscopy, scanning probe microscopy (SPM), such as atomic force microscopy (AFM)^21,22^ and scanning tunneling microscopy (STM),^23,24^ X-ray absorption spectroscopy (XAS),^25,26^ X-ray photoelectron spectroscopy (XPS),^27^ scanning electrochemical cell microscopy (SECCM),^28–30^ surface-enhanced Raman spectroscopy (SERS),^31^ and TERS.^32^ *In situ* electron microscopy, especially *in situ* transmission electron microscopy (TEM),^33^ has been developed for studying the structural evolution of solid materials during catalytic reactions in liquid.^34–36^ Similarly, SPM can provide surface morphology and electronic structure at the atomic resolution under various measurement conditions.^37,38^ However, these methods provide limited chemical information on the surface of catalysts. Recently, X-ray microscopies, such as ambient pressure XPS and XAS, have shown great potential in revealing the chemical composition and the electronic structure of the surfaces as well as the surface species.^39,40^ Nevertheless, the *in situ* studies of X-ray microscopies in liquid are still challenging, for which purpose an *in situ* reactor is required. Among these techniques, TERS stands out for its ability to simultaneously provide geometric and chemical information on the surface of catalysts at the nanoscale, and for its easy implementation under various conditions, including ultrahigh vacuum, air, and liquid (under electrochemical control).

Raman spectroscopy provides chemical fingerprint information on molecules.^41,42^ However, the small Raman cross-section of molecules usually renders this method unsuitable for studying monolayers. Another bottleneck is the spatial resolution, which is restricted by the diffraction limit.^23^ TERS was first demonstrated in 2000 ^43–46^ and has rapidly developed into a powerful tool for the chemical analysis of surfaces over the past two decades.^47–53^ TERS couples SPM with plasmon-enhanced Raman spectroscopy, and thus combines subnanometer spatial resolution (under ultrahigh vacuum and cryogenic conditions) with single molecule sensitivity.^54^ In this method, a Ag or Au tip with a radius of approximately 20 nm is commonly used as a probe. The plasmonic tip is brought onto the sample surface with a distance below 1 nm using the feedback loop of the SPM. Under the resonant illumination of the tip apex with a laser, plasmons induced at the gap between the tip and the substrate result in a highly localized electromagnetic field, which enhances the Raman signal of the molecules under the tip.^55^ Due to the strong localization of the enhanced electromagnetic field at the tip apex, TERS can probe the topographic and chemical structure of the sample with a spatial resolution of approximately 2–3 nm at ambient conditions.^56–58^ Under ultrahigh vacuum (UHV) and cryogenic conditions, TERS can even achieve spatial resolution down to the Angstrom level,^59–61^ which is rationalized by the ultimate confinement of light at the plasmonic picocavity in the junction.^62–64^ This ability allows for a direct correlation between the specific surface structure and the catalytic performance in a label-free and nondestructive way, revealing catalyst–molecule interactions during heterogeneous catalysis. Furthermore, TERS can be effectively implemented in ambient, liquid, or even electrochemical environments, which makes it a feasible tool for operando monitoring of catalytic reactions.^65–70^

This review summarizes recent advances in the application of TERS for heterogeneous catalysis and highlights some key studies where TERS has been employed to probe the model catalysts, focusing on critical aspects such as active sites, molecular activation pathways, conversion efficiency, chemical selectivity, and operando studies. We then provide a detailed discussion on mechanistic insights into catalysis revealed by TERS across various model systems. Finally, we outline current challenges and potential strategies for advancing TERS studies in heterogeneous catalysis, concluding with future directions for developing TERS in this field.

## 2 Critical Insights into Catalytic Reactions

### 2.1 Active Sites

Studying the physicochemical properties of surface active sites of catalysts at the molecular level can provide valuable insights into the relationship between the structures of catalysts and their catalytic performance.^71^ In heterogeneous catalysis, the solid catalyst provides the surface sites for the adsorption and reaction of molecules, along with the generation of intermediates and desorption of products. Some minor surface sites, including step edges, defects, and perimetrical interfaces of various catalytic materials, are often highly active in heterogeneous catalysis. It is still challenging to visualize active sites with nanoscale spatial resolution under catalytically relevant conditions. Recently, TERS has been applied to monitor the catalytic activities of different surface sites of model catalysts. TERS is ideally suited for this work, as it offers high spatial resolution down to a few nm under ambient conditions, making it highly effective for revealing surface active sites.^58^

In 2015, Kumar et al. reported the first TERS study of the catalytically active sites of a Ag substrate for the plasmon-induced reactions of *para*-aminothiophenol (*p*ATP) to *p*,*p*′-dimercaptoazobenzene (DMAB).^72^ An aluminum-coated AFM tip was employed to enhance the Raman signal, meanwhile, the direct photocatalytic reactions at the tip apex were blocked. TERS maps reveal that the photocatalytic reactions only occurred at certain locations of the Ag substrate, evidenced by the distribution of the Raman peaks of DMAB. The catalytically active and inactive sites were mapped with a spatial resolution of 20 nm. Due to the complicated catalyst structure, it is still challenging to establish a full understanding of the catalytic processes. To simplify the geometric structure of solid catalysts, the Ren group fabricated a well-defined bimetallic model catalyst using underpotential deposition (UPD).^58^ In this way, they prepared a submonolayer of Pd on a Au(111) surface. The phenyl isocyanide (PIC) molecule was used as a probe molecule because its NC triple bond can directly interact with the surface and is sensitive to the electronic property of the surface, which allows us to distinguish different local structures of catalysts. TERS results showed that the N≡C vibrational peak gradually decreased and the peak at 1590 cm^–1^ significantly broadened on Au(111), while these changes were not observed on the Pd surface. This indicates that PIC is oxidized to phenyl isocyanate on Au(111), but surprisingly not on the Pd surface. Further, TERS line scans across a Pd–Au–Pd area showed a stronger TERS signal of PIC due to a stronger electromagnetic field located at the Pd step edges, with 3 nm spatial resolution (Figure 1a,b). Moreover, a lower frequency band at 1933 cm^–1^ was observed at the Pd step edge (Figure 1c), which is attributed to the weakened vibration of the NC triple bond of PIC molecules adsorbed. This result suggests an enhanced oxidation reactivity of PIC molecules at the Pd step edge compared to the Pd terrace, due to the d-band shift of the low-coordinated Pd step edge atoms to higher energy. Step edges of the Pd surface possess higher activity than the terrace. This is the first report to probe the oxidation of PIC molecules on metal surfaces using ambient TERS, achieving an exceptionally high spatial resolution of ∼3 nm. The authors effectively show the temporal evolution of TERS spectra for PIC on both Au and Pd. However, a missing element is deeper insight into the reaction dynamics at the Pd/Au step edges, likely due to the sluggish oxidation rate at these specific sites. Likewise, the Zenobi group demonstrated a higher *trans*-to-*cis* photoisomerization efficiency of azobenzenethiol at the Au step edges than that on the Au terraces. DFT suggests that it is also ascribed to the shift of d-bands to higher energy and the lower reaction enthalpy of *trans*-to-*cis* isomerization at Au steps.^48^ Additionally, the Kurouski group found that the step edges and corners of Pd on Au bimetallic nanoplates are the active sites for the Pd-catalyzed Suzuki–Miyuara reaction, rather than the Pd terrace, demonstrated with cargo-TERS.^70^ These studies demonstrate that step edges and corners, due to their distinct electronic structures, exhibit stronger catalytic activity than terraces, which is a consensus in heterogeneous catalysis. For the plasmon-induced dimerization of 4-bromothiophenol on nickel-decorated Au nanoplates, they discovered that it primarily occurred on Ni nanoislands rather than the surrounding Au, which demonstrated that Ni islands served as active sites during the catalytic reaction.^73^

**Figure 1:**
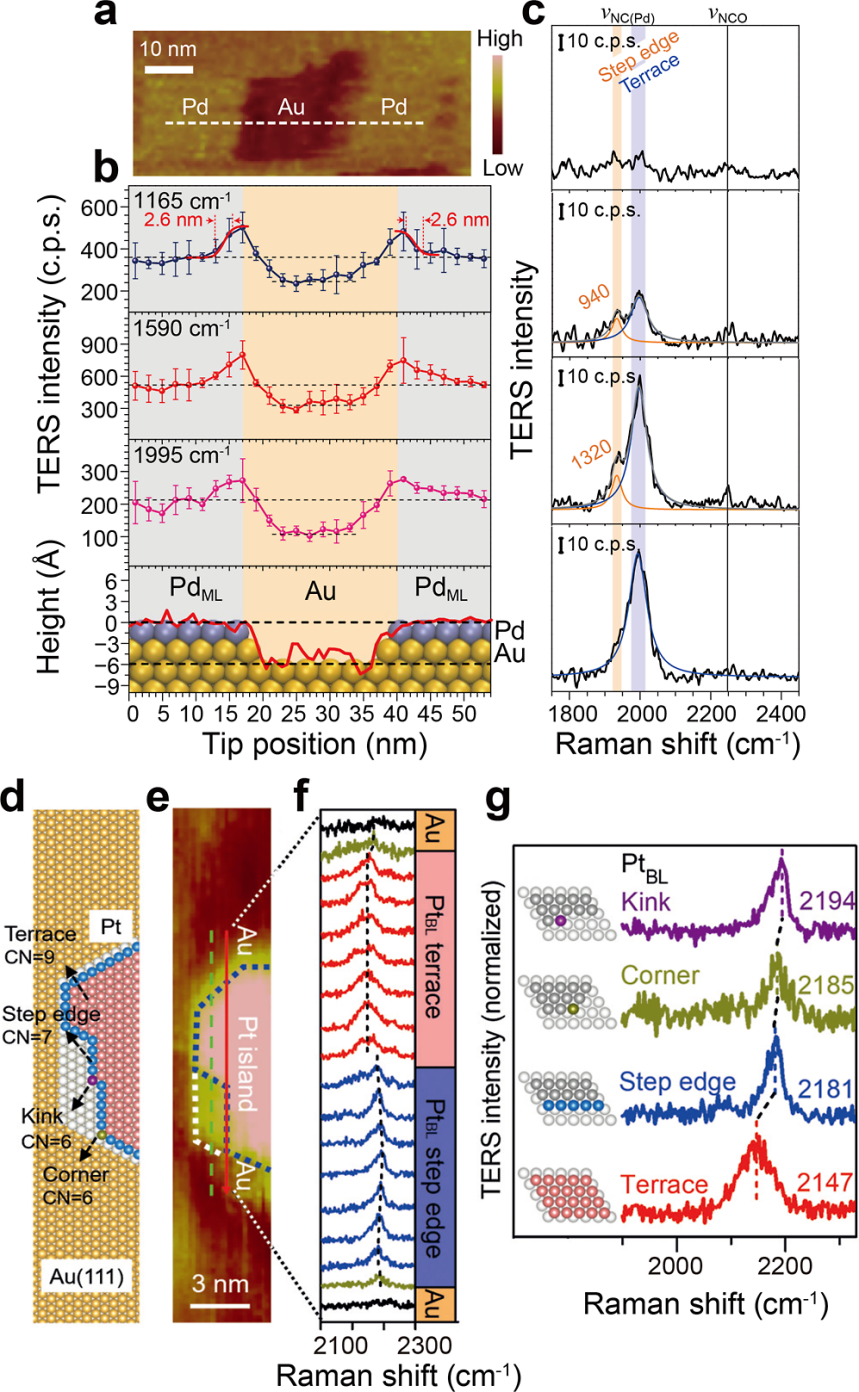
Relationship between the catalyst structure and catalytic activity. (a) STM image of the Pd submonolayer on a Au(111) surface with adsorbed PIC, showing a gold hole in the middle. (b) Top three panels: plots of intensities of the three main TERS peaks (1165, 1590, and 1995 cm^–1^) as a function of the tip position. Error bars indicate the standard deviation for the three measurements. Bottom panel: Height profile of the surface along the dashed line in (a) superimposed with the atomic model of the surface atoms. The adsorbed PIC molecules on the surface are not included in the model. (c) Line-scan TERS spectra (from bottom to top) across a Pd terrace-Pd step edge-Au terrace region. Reprinted and adapted with permission from ref 58 Copyright 2017 Springer Nature. (d) Atomic model of a Pt nanoisland on Au(111) shown in (e). Yellow, Au; white, first-layer Pt; red, blue, purple, and dark yellow, second-layer Pt. (e) STM image of the Pt nanoisland on the Au(111) surface. (f) TER spectra acquired along the red solid line in (e). (g) Representative TER spectra of the *v*_NC_ peak of CPI adsorbed at different atomic sites of Pt. Reprinted and adapted with permission from ref 16 Copyright 2018 John Wiley and Sons.

The local electronic property of solid catalysts, such as the coordination environment, can efficiently tune the interfacial metal/molecule electronic structure and thus the charge transfer kinetics. Su et al. fabricated Pt nanoislands on Au(111) through UPD of Cu on a Au(111) surface, followed by galvanic replacement of Pt. 4-Chlorophenyl isocyanide (CPI) was used as a probe molecule.^16^ In Figure 1d–g, a clear shift of the N≡C vibrations of CPI is observed when the tip crosses over the Pt terrace to the step edge, and to the kink. DFT confirms that a lower coordination number at the step edge and kink than that on the Pt terrace leads to a higher d-band center, resulting in stronger metal/molecule interaction and thus the shift of the NC peak of CPI molecules to a higher vibrational frequency. This work is strong evidence that TERS is well suited for the *in situ* studies of the various surface sites and their catalytic properties of solid catalysts.

Furthermore, the distribution of active sites plays a key role in determining the local catalytic performance of catalysts. Ex situ TERS studies provide a straightforward and efficient approach to observe the molecular changes after catalytic reactions, which can offer direct evidence for the identification of catalytically active sites. The hydrogen spillover depicts the dynamic migration of surface adsorbed hydrogen species from hydrogen-rich sites to hydrogen-poor sites, which is an important effect in H-involving reactions.^74^ Unveiling how hydrogen transfers on the surface of solid catalysts is essential for enhancing the catalytic performance of H-involving reactions, which is, however, hampered due to the structural complexity of powder catalysts, especially for oxide catalysts.^75^ Recently, Yin et al. employed TERS to study the spatial distribution of active hydrogen atoms and their catalytic activities, thereby elucidating the structure–reactivity relationship on the surface of Pd/Au bimetallic model catalysts.^76^ The hydrogenation of chloronitrobenzenethiol (CNBT) was used as the model system, utilizing the catalytic reduction of CNBT to chloroaminobenzenethiol (CABT) in the presence of hydrogen to identify the catalyst’s active sites (Figure 2a). TERS maps demonstrated that CNBT is selectively hydrogenated to CABT on the Pd surface, revealing a clear correlation between the location of the Pd area and the spatial distribution of CABT in Figure 2b. Moreover, a slight discrepancy between the spatial distribution of CABT and Pd was observed. The TERS intensity of the NO_2_ vibrational peak at 1336 cm^–1^ even decreases in the Au crater, indicating the reduction of the nitro group of CNBT on Au. By calculating the adsorption energy of species on Pd and Au, they concluded that this discrepancy is attributed to hydrogen spillover, where hydrogen molecules dissociate on Pd initially and then hydrogen atoms diffuse to the adjacent Au surface, facilitating the reduction of CNBT on Au. To gain a further understanding of the hydrogenation process of CNBT, a quantitative characterization of the relationship between the active sites (blue region in Figure 2c) and surface structure was conducted. The colocalized STM image with the height profile is also given (Figure 2d), showing a Pd island on a Au surface. For the Au surface with low Pd coverage, the size of the active region is approximately 50 nm,^76^ while the size of the Pd island is only 20 nm. With more TERS results, they found that the reactive regions are ∼15–30 nm larger than the Pd areas, indicating that hydrogenation occurs beyond the Pd active sites and onto the Au areas. This work investigated the hydrogenation of the CNBT self-assembled layer on Pd/Au bimetallic model catalysts using TERS with approximately ∼10 nm chemical spatial resolution, enabling the visualization of catalytically active sites. Since the hydrogenation products on Pd and Au are identical, it is difficult to visually track the hydrogen spillover. A better model reaction would yield distinct products at different sites, for example, hydrogenation on Pd and dehalogenation on Au (within the spillover region). This would allow the spillover region to be easily identified in TERS maps by locating the dehalogenation products.

**Figure 2:**
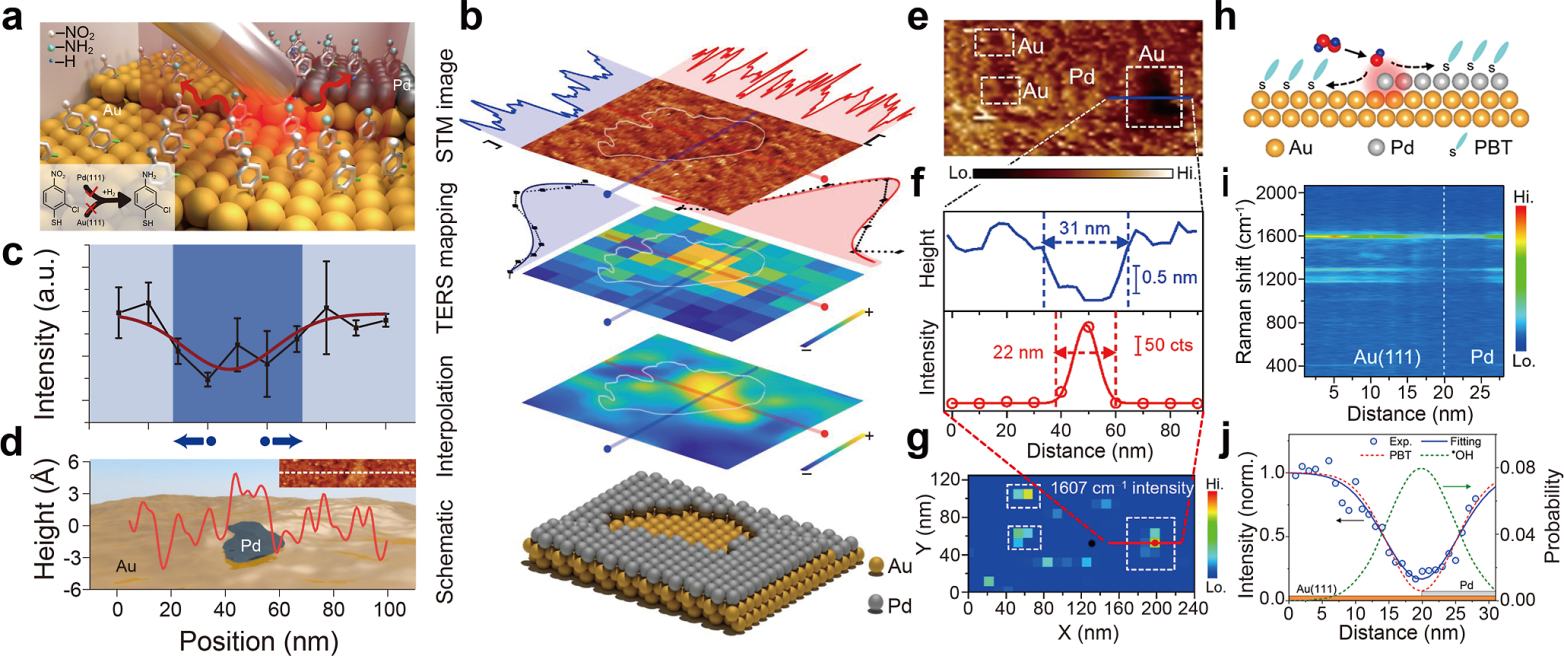
Catalytically active sites and the diffusion range of reactive species revealed by TERS. (a) Scheme of the selective catalytic hydrogenation of a CNBT self-assembled monolayer (SAM) on a Pd/Au bimetallic surface. (b) First panel: STM image of CNBT SAM on submonolayer Pd on Au(111) after exposure to H_2_, showing a Au crater on the Pd surface. Second panel: the normalized TERS map of the peak at 1336 cm^–1^ (NO_2_ stretching mode) of CNBT SAM on high coverage Pd on Au(111) after exposure to H_2_, colocalized with the STM image in the first panel of b. Third panel: Cubic spline interpolation of the TERS map shown in the second panel of b. Fourth panel: atomic model of a Au crater on the Pd surface. (c) Plot of the TERS intensity of the peak at 1336 cm^–1^ in Raman spectra in TERS line scan spectra on low Pd coverage on Au(111) after exposure to H_2_ as a function of the tip position. The size of the active region defined by the peak width of the fitted curve (red solid line) is represented by the dark blue region. Blue arrows accompanied by dots indicate the hydrogen spillover direction. (d) Height profile (red line) of the surface along the dashed line of the inset of the corresponding STM images, superimposed with a schematic of the surface structure in (d). Reprinted and adapted with permission from ref 76 Copyright 2020 Springer Nature. (e) STM image of a PBT SAM on a Pd/Au(111) bimetallic surface after reaction in 30% H_2_O_2_ aqueous solution. (f) Height profile (top panel) along the blue solid line in (e) and TERS intensity profile (bottom panel, red circle) along the red solid line in (g). (g) Corresponding 1607 cm^–1^ TERS peak map recorded simultaneously with the STM image in (e), using the intensity of the peak at 1607 cm^–1^. (h) Schematic illustration of the diffusion of OH radicals generated at Pd step edge active sites on a Pd/Au(111) bimetallic surface. (i) Waterfall plots of all TER spectra from a TERS line scan. Step size: 1 nm. The vertical dashed line indicates the position of the Pd step edge. (j) TERS intensity profiles (blue dots) of the 1607 cm^–1^ peak in (i) fitted with a Gaussian function (blue line). The spatial distribution of the number of unreacted PBT molecules obtained by deconvolution (red dashed curve). The diffusion-induced distribution of the probability of OH radicals (green dashed curve). Reprinted and adapted with permission from ref 77 Copyright 2020 American Chemical Society.

Similarly, Su et al. applied TERS to investigate the local generation and diffusion of active oxygen species (AOS) on a Pd/Au bimetallic model catalyst.^77^ They used 4′-(pyridin-4-yl)­biphenyl-4-yl)-methanethiol (PBT) as a Raman marker. TERS maps revealed that PBT underwent oxidative degradation by AOS which is generated on the Pd surface after immersion in 30% H_2_O_2_ solution for 30 min, resulting in the disappearance of PBT Raman signal (Figure 2e–g). They discovered that H_2_O_2_ can only be activated to generate highly active OH radicals on the Pd surface but not on the Au surface. These OH radicals subsequently destroyed the adsorption capacity of PBT on the metal surface, reducing the Raman signal intensity of PBT on the catalyst surface. However, TERS maps showed that the size of the area with a positive PBT signal was around 9 nm smaller than that of the Au-hole region, indicating that OH radicals diffuse to the adjacent Au area upon generation. To distinguish the activity between different Pd sites, the reaction was conducted under mild conditions (immersion in 15% H_2_O_2_ solution for 2 min). It is found that PBT signals near the Pd step regions decreased, while the TERS intensity of PBT at the Pd terrace remained comparatively strong, similar to that observed on the Au(111) surface (Figure 2h–i). This indicated that the Pd step edge is more active than the Pd terrace in generating AOS. Gaussian function deconvolution of the TERS signal of PBT at the Pd/Au interface indicated that the OH radical diffusion length at the Pd step edge was approximately 5.4 nm (Figure 2j). These results provide valuable insights into the spatial distribution of the active sites in AOS-induced catalytic reactions promoted by Pd and demonstrate the spillover of AOS.

### 2.2 Molecular Activation Pathway

Gaining insight into the molecular activation pathway at the nanoscale to unveil catalytic mechanisms is crucial for developing highly efficient catalysts. The challenge of distinguishing individual molecules and their interaction with metal surfaces can be overcome by employing analytical techniques with ultrahigh spatial resolution, such as STM and TERS, to achieve molecular-level imaging of surfaces. Compared with STM, TERS has an inherent advantage as it provides the chemical information on the surface simultaneously with the topography. Thus, it has been widely applied to study the chemical reaction processes of surface molecules, such as coupling,^52,78,79^ decomposition,^60,80,81^ and oxidation.^82–84^

Recently, TERS was employed to investigate the oxidation of ordered and disordered *p*ATP monolayers on Au by oxygen and elucidate the oxygen activation pathway on gold surfaces^83^ (Figure 3a). To gain a more comprehensive understanding of the oxidation of the ordered and disordered *p*ATP on Au, a quantitative analysis of the conversion efficiency of *p*ATP to *p*-nitrothiophenol (*p*NTP) after exposure to oxygen was performed. In Figure 3d,e, TERS intensity maps of the NO_2_ group demonstrate that the disordered *p*ATP monolayer on Au shows a higher reaction efficiency compared to that on the ordered SAM sample (Figure 3b,c), after exposure to O_2_. Direct oxidation of *p*ATP adlayers in H_2_O_2_ solution affirms that the oxidation of *p*ATP molecules proceeds via interaction with on-surface oxidative species. When *p*ATP molecules were arranged in an ordered phase on the gold surface, spatial constraints limited their interaction with reactive oxygen species, inhibiting the oxidation reaction. The detailed activation pathway of O_2_ on Au surfaces was further demonstrated by the isotope labeling method. The observed 3 cm^–1^ red shift of the NO_2_ Raman peak during *p*ATP oxidation with H_2_^18^O (Figure 3d) is unexpectedly small. Isotope labeling with ^18^O typically produces shifts of an order of magnitude larger (tens of cm^–1^).^85–87^ The minimal shift here indicates that only a tiny amount of the N^18^O_2_ group is formed, implying that oxygen activation via interfacial water on the Au surface is a limited process. Nevertheless, this result provides empirical evidence for the generation of active oxidative species, such as hydroperoxyl radicals, atomic oxygen, and hydroxyl radicals (Figure 3e), on the Au(111) surface through a water-promoted O_2_ activation mechanism. This study reveals the role of water in promoting oxygen activation using TERS, promoting the fundamental understanding of oxygen activation mechanisms on gold surfaces. These findings provide strong support for the application of TERS in elucidating molecular activation pathways on catalytic surfaces. Despite this advance, what is missing in the report is to provide the spectroscopic evidence of these active oxidative species induced by O_2_ and interfacial water using TERS, which will help gain deep insights into the molecular activation process on metal substrates.

**Figure 3:**
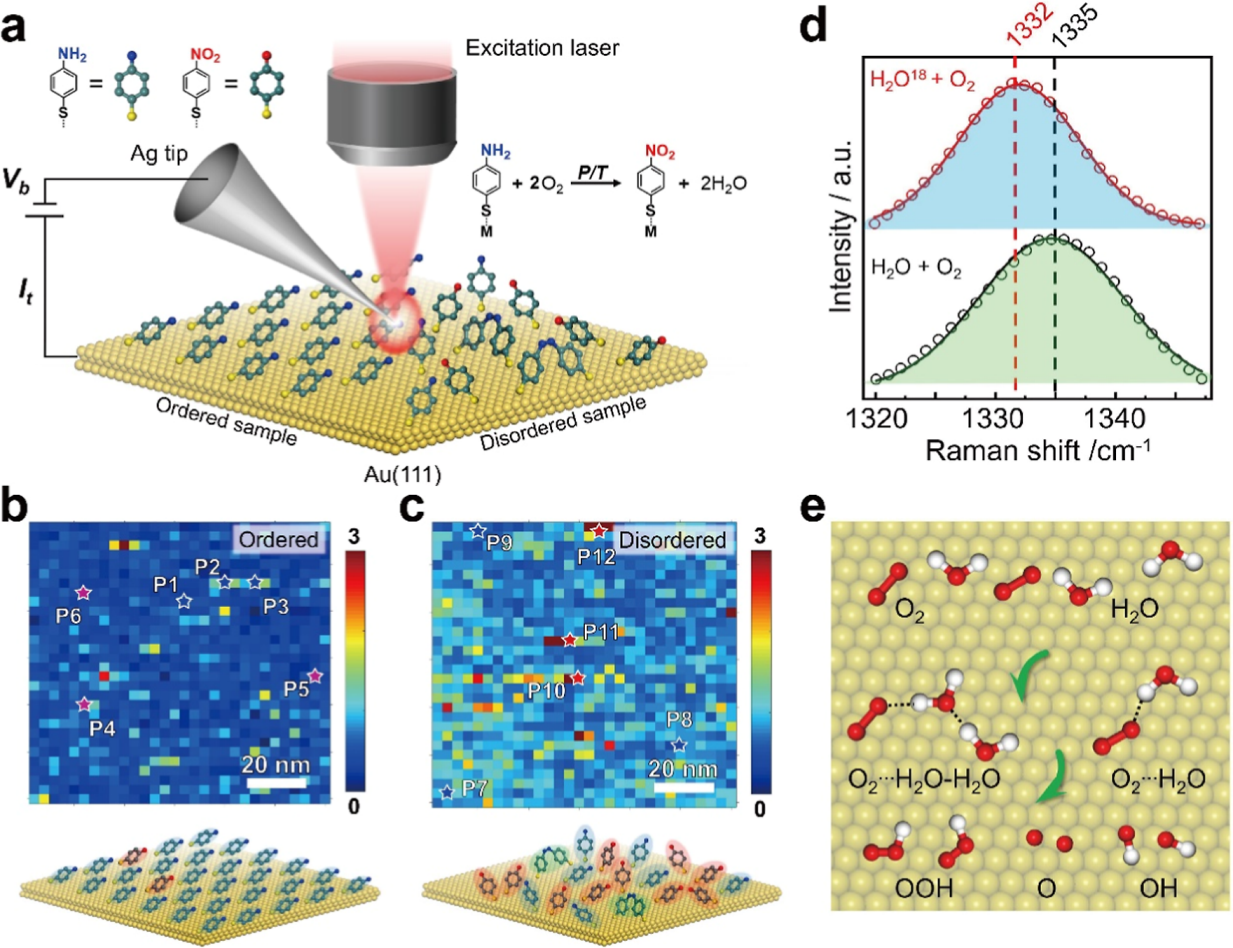
Insights into molecular activation pathways at the nanoscale via TERS. (a) Schematic of studying the oxidation of the ordered and disordered *p*ATP on Au using STM-TERS. (b,c) Top panels: TERS intensity (1335 cm^–1^ peak) maps of the ordered (b) and disordered (c) *p*ATP samples after O_2_ treatment. Bottom panels: the corresponding atomic models for illustration. (d) The average TERS spectra of the H_2_^18^O-treated *p*ATP adlayer and the *p*ATP adlayer without H_2_^18^O treatment after O_2_ exposure. (e) Schematic illustrating the theoretically proposed water-promoted mechanism of O_2_ activation on Au(111) surface. Reprinted and adapted with permission from ref 83 Copyright 2024 John Wiley and Sons.

### 2.3 Conversion Efficiency

Conversion efficiency of catalysts is one of the important parameters to evaluate the chemical performance of the solid catalysts, which is determined by various factors, including the composition of catalysts, the orientation of adsorbed molecules, and the reaction time. Taking advantage of the high sensitivity of TERS, one can (sub)­quantitatively evaluate the conversion efficiency of solid catalysts in catalytic reactions. TERS combined with theoretical calculations provides fundamental aspects in catalysis to unravel the underlying catalytic mechanism.

Plasmon-driven photocatalytic coupling reactions, particularly the oxidation of *p*ATP and the reduction of *p*NTP to form DMAB, have emerged as prominent model systems in the SERS and TERS fields due to their utility in studying photocatalytic mechanisms and plasmonic enhancement effects.^88^ In 2012, van Schrojenstein Lantman et al. pioneered the study of TERS to investigate the photocatalytic coupling reaction of *p*NTP to form DMAB on a gold substrate.^65^ The study employed a dual-wavelength laser strategy: a 532 nm (green) laser triggered the photocatalytic coupling reaction of *p*ATP, while a 633 nm (red) laser monitored its progression in real time, because the coupling process was selectively activated by green light.^89^ This approach enabled the direct observation of the reaction kinetics of *p*NTP dimerization at the nanoscale. Furthermore, the study demonstrated that the reaction rate was significantly influenced by the surface coverage of *p*NTP, with partially covered regions exhibiting a faster reaction rate compared to fully covered monolayers. Recently, the Kurouski group investigated this catalytic reaction on tungsten disulfide (WS_2_) nanoplates supported on a Si substrate.^90^ They found that the catalytic activity was significantly enhanced when the WS_2_ nanoplates were functionalized with Pd nanoparticles. This study highlights the potential of coupling catalytic metals with transition metal dichalcogenides to enhance their catalytic performance. However, coupling two-dimensional materials with metal nanoparticles yields a complex with complicated structure, making it difficult to disentangle the catalytic mechanisms on the complex. Beyond surface coverage, the molecular orientation of *p*NTP on gold surfaces also plays a crucial role in determining the reaction rate. Cai et al. employed TERS mapping to investigate the impact of ordered and disordered molecular arrangements on the dimerization of *p*NTP to form DMAB (Figure 4a).^78^ To this end, two different sample preparation methods were used. The ordered *p*NTP SAM was prepared by immersing the Au substrate in a *p*NTP solution overnight. This method resulted in well-organized molecular domains with sizes of tens of nanometers, as shown in Figure 4b. In contrast, the disordered *p*NTP sample was fabricated via drop-casting of *p*NTP onto Au. This rapid deposition method produced only sparse molecular domains with sizes of a few nanometers, as observed in Figure 4c. The TERS peaks at 1146, 1390, and 1442 cm^–1^ (corresponding to DMAB vibrational modes) exhibit significantly stronger intensities in the disordered *p*NTP samples compared to the ordered SAMs (Figure 4d,e). The enhanced signal suggests that the varied molecular orientations of *p*NTP in disordered configurations promote a higher conversion efficiency for *p*NTP dimerization into DMAB. DFT calculations further demonstrated that in the disordered sample, *p*NTP molecules exhibit greater orientational flexibility, resulting in a lower reaction energy barrier. Similarly, the Ren group found that the plasmon-induced dimerization of *p*ATP on Au(111) and on Ag(111) is highly dependent on the adsorbed molecular orientations.^91^ In Figure 4f, the Raman peaks of DMAB appeared in the TERS spectrum of *p*ATP SAM on Au(111) following 5 s irradiation of a 633 nm laser. In contrast, no DMAB Raman peaks were detected in the TERS spectrum of *p*ATP SAM on Ag(111) even after 10 min of irradiation with the same wavelength (Figure 4g). DFT simulations revealed that the most stable configuration of *p*ATP on Au(111) occurred with the molecular axis tilted 31° relative to the normal surface (Figure 4h), while the tilt angle of *p*ATP on Ag(111) corresponding to its optimized configuration was only 16°. This suggests that *p*ATP molecules adopt a more vertical orientation on Ag(111), thereby inhibiting their dimerization. Furthermore, the authors demonstrated that *p*ATP coupling reactions could occur on polycrystalline Ag substrates, confirming that molecular orientation variations facilitate this reaction. Notably, the coupling reaction of *p*ATP is also dependent on the metal substrates, for example, it can take place on Au(111) but not on Au(100).^92^ By correlating molecular orientation with reactivity, both studies independently demonstrated that adsorption geometry is a critical factor controlling the efficiency of plasmon-driven catalytic reactions.

**Figure 4:**
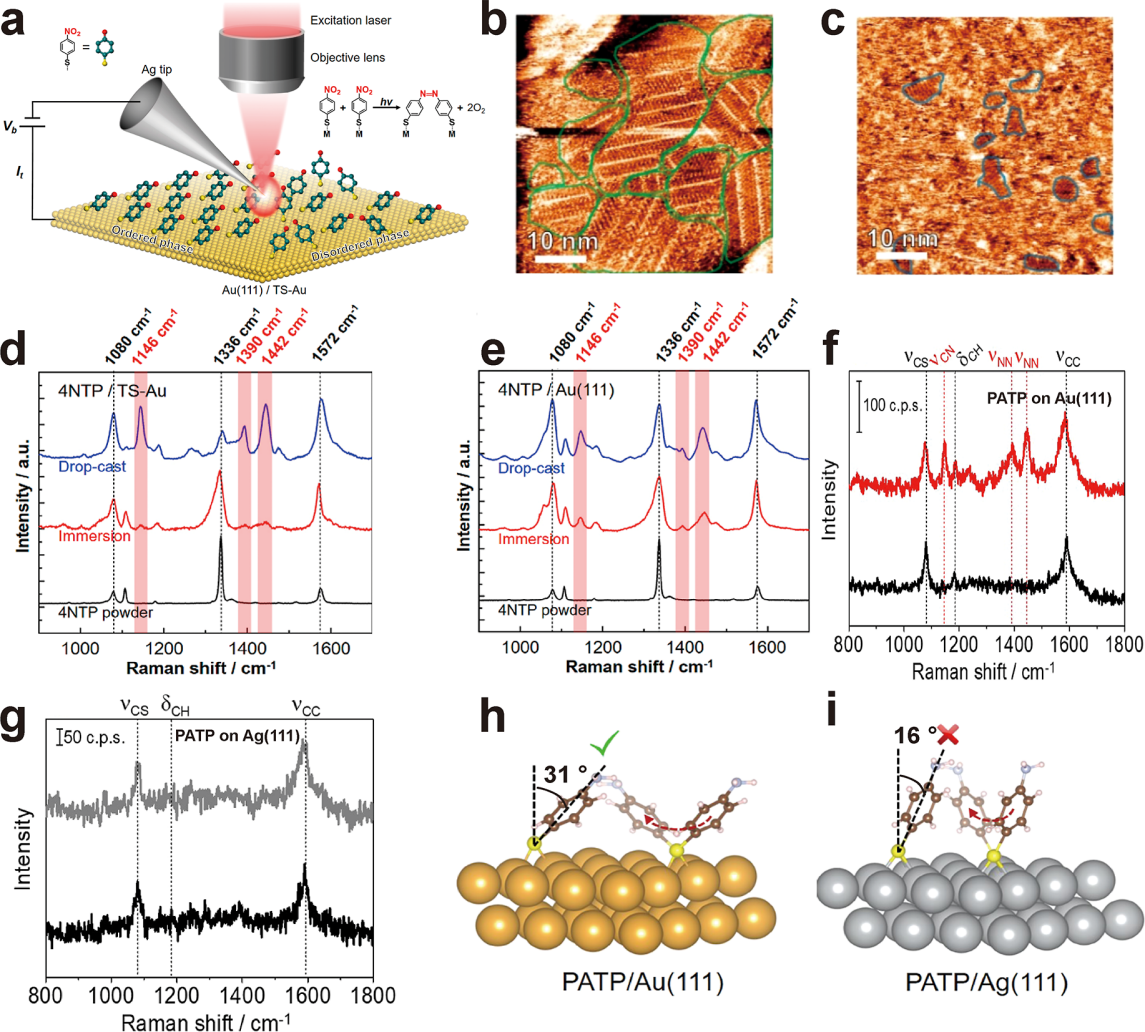
Correlation between conversion efficiency and molecular orientation on the surface. (a) Schematic of the TERS measurement of ordered and disordered *p*NTP to form DMAB on a Au surface. (b,c). STM images of the *p*NTP adlayers on Au prepared via immersion (b) and drop-cast protocols (c). (d,e) Average TERS spectra of *p*NTP-functionalized (d) TS-Au and (e) Au(111) surfaces prepared via drop-cast (blue trace) or immersion (red trace) protocols and the confocal Raman spectrum of *p*NTP powders (black trace). Reprinted and adapted with permission from ref 78 Copyright 2022 American Chemical Society. (f,g) Top panels: STM images of *p*ATP adlayers on a Au(111) surface (f) and a Ag(111) plate (g). Bottom panels: The corresponding profiles along the white dashed lines indicated on the top panels of Figure 4f,g. (h,i) TERS spectra acquired on *p*ATP SAMs on Au(111) (h) and Ag(111) (i) substrates marked with the white dots in the top panels of Figure 4f,g. The black spectra were collected before the laser irradiation. The red spectrum in Figure 4h and the gray spectrum in Figure 4i were collected after 5 s (h) and 10 min (i) of laser irradiation, respectively. (j,k) Atomic models illustrating the optimized molecular arrangements of *p*ATP on Au(111) (j) and Ag(111) (k). Reprinted and adapted with permission from ref 91 Copyright 2019 American Chemical Society.

### 2.4 Chemical Selectivity

Chemical selectivity in heterogeneous catalysis is a cornerstone of catalytic performance and is inherently linked to the catalyst’s microstructure dictating reactant adsorption, activation, and reaction pathways. TERS offers transformative insights into these phenomena by combining ultrahigh spatial resolution and single-molecule sensitivity. These capabilities allow TERS to probe catalytic selectivity at the single-bond level, resolving localized chemical interactions and transient intermediates that are otherwise obscured in the ensemble-averaged techniques. In heterogeneous catalysis, chemical selectivity refers to the ability of a catalyst to direct a chemical reaction toward a specific desired product(s) over other thermodynamically feasible products. Selective chemical bond activation (bond selectivity within a single molecule) is a crucial manifestation of reaction selectivity. We will use a few recent studies, including selective bond breaking within a single molecule directed either by plasmon or by voltage pulses in STM, and selective product formation controlled by metals (reaction selectivity), to exemplify the chemical selectivity studied by TERS.

Although chemical reactions assisted by surface plasmons have been extensively studied in the past decades, the precise control of localized plasmons to activate a specific moiety of a molecule, in the presence of multiple chemically equivalent parts within a single molecule, is extremely challenging due to the relatively large lateral distribution of the plasmonic field. In a recent study, Mahapatra et al. demonstrated the selective activation of a C–Si bond in individual 5, 10, 15, 20-(tetra-trimethylsilylethynyl) porphyrin (TMSEP) molecules on Cu­(100).^93^ Under UHV and cryogenic conditions, they combined STM with light irradiation to achieve this with single-bond precision (Figure 5a,b). Atomic-scale confinement of the localized surface plasmon, which was achieved by holding the STM tip within a subnanometer proximity to the target molecule for several seconds under laser excitation, facilitated the selective bond breaking. The authors demonstrated the sequential cleavage of the three C–Si bonds within a single TMSEP molecule, leading to the formation of carbon radicals presenting a different height contrast in STM images (Figure 5c–f). The resultant carbon radical intermediates exhibited characteristic topographic variations in STM imaging, manifesting as distinct height contrast features that provided direct spatial evidence of the bond-breaking sequence. This work is among the few to successfully demonstrate plasmon-induced chemistry at the single-bond level.

**Figure 5:**
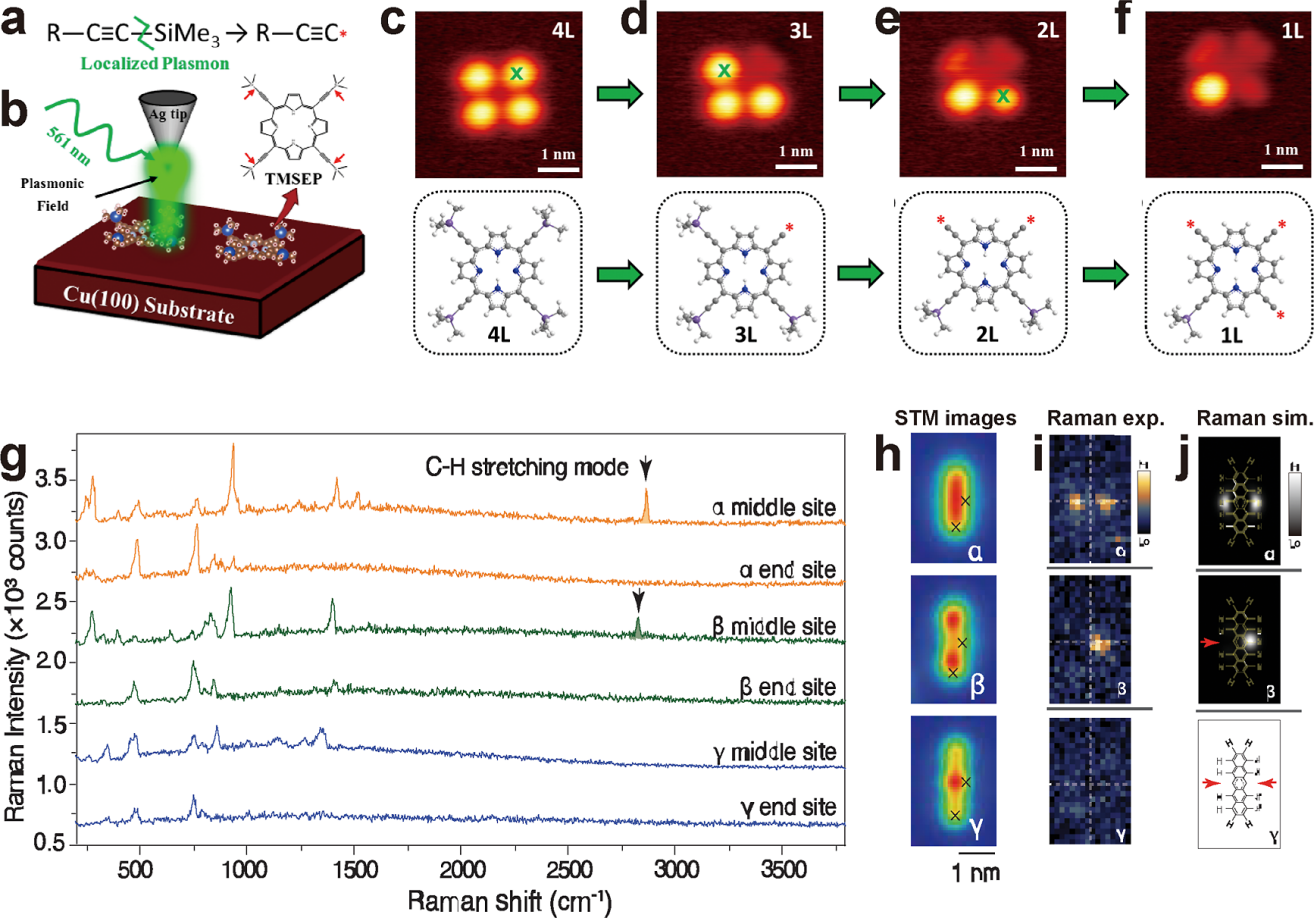
Chemical selectivity at the single bond level. (a) Simplified scheme of the C–Si bond breaking. (b) Schematic of the plasmon-assisted site-selective activation of a single TMSEP molecule on Cu(100) in the STM nanojunction by a Ag tip under the illumination of a 532 nm laser. (c–f) Consecutive C–Si bond dissociations within a TMSEP molecule. Top panel: STM image of TMSEP with 4 lobes (c), 3 lobes (d), 2 lobes (e), and 1 lobe (f), respectively. Bottom panel: a corresponding ball-and-stick model of the molecule. Reprinted and adapted with permission from ref 93 Copyright 2022 American Chemical Society. (g) TER spectra obtained at the middle and end sites indicated by the crosses on the pentacene species of α, β, and γ in panel h. Excitation source: 532 nm with an intensity of 0.2 mW. Exposure time: 5 s. The spectra are vertically shifted for clarity. (h) STM images of the pentacene species of α, β, and γ. The transformed species (β, and γ) were achieved by voltage pulses. Tunneling conditions: *V* = 0.1 V and *I* = 8 nA. (i). TERS maps of the C–H stretching mode of the pentacene species α, β, and γ. (j). Simulated Raman maps of the C–H stretching mode of the three pentacene species. Red arrows indicate the C–H bond breaking at the central benzene ring in β and γ. Reprinted and adapted with permission from ref 60 Copyright 2021 American Association for the Advancement of Science.

Similarly, Xu et al. utilized a combined STM, AFM, and TERS to characterize the structure of individual pentacene molecules on Ag(100) under UHV and cryogenic conditions.^60^ They found that pentacene molecules (intact, α) can be selectively transformed to derivatives (β and γ) through the specific C–H bond breaking driven by the pulsed voltage or more possibly by tip-induced nanocavity plasmons, which is deduced by the same voltage threshold for both pathways at 1.5–1.6 V (Figure 5h). For the detailed mechanism of the bond breaking, the readers are referred to the ref 60. TERS was employed to unravel the chemical structure of the three species. Figure 5g shows the TER spectra taken from the species α, β, and γ when the tip was located around the middle and end sites of the molecules. For species α and β, the Raman peak at around 2850 cm^–1^ corresponding to the C–H stretching mode is visible when the tip is placed in the middle of the molecule, while for species γ, this peak completely disappears. Furthermore, TERS mapping shows the spatial distribution of the C–H vibrational mode, as confirmed by DFT simulations (Figure 5i,j). The structure of the three species was also investigated by AFM imaging. The combined STM-AFM-TERS strategy enabled the unambiguous correlation between the structure and chemical heterogeneities of the three pentacene-derivative species obtained by the specific C–H bond breaking, which demonstrated the precise control of single bond selectivity at the atomic level.

Bimetallic nanostructures composed of plasmonic and catalytic metals exhibit unique catalytic reactivity and selectivity in heterogeneous catalysis, especially in plasmon-driven reactions. Recently, the Kurouski group used TERS mapping to investigate the plasmon-induced redox chemistry of 4-mercaptophenylmethanol (MPM) and 4-mercaptobenzoic acid (MBA) on gold–platinum bimetallic nanoplates (Au@PtNPs), gold–palladium bimetallic nanoplates (Au@PdNPs), and their monometallic counterparts, gold nanoplates (AuNPs).^94^ TERS mapping revealed distinct catalytic behaviors for MPM on Au@PtNPs and MBA on Au@PdNPs. On Au@PtNPs, a new peak at 1714 cm^–1^ (assigned to the C=O vibrations) appeared predominantly in the spectra from the edges of the nanoplates (Figure 6b–e), confirming the plasmon-driven oxidation of MPM to MBA at these sites. No evidence of the reverse reaction (MBA reduction to MPM) was observed on Au@PtNPs, indicating that the oxidation was exclusive to the edges of Au@PtNPs (Figure 6a). In contrast, Au@PdNPs facilitated only the plasmon-driven reduction of MBA to MPM, as shown in TERS maps (Figure 6f–h). These results highlight that the reactivity and selectivity of bimetallic nanoplates depend critically on the catalytic metal. Kinetic studies further revealed that the reduction rate on Au@PdNPs exceeded the oxidation rate on Au@PtNPs. Notably, monometallic AuNPs exhibited different selectivity: both MPM and MBA were converted to thiophenol (TP) (Figure 6i–m), underscoring the unique catalytic properties of bimetallic systems. Their findings suggest that while the rectified electric field likely serves as the driving force, the catalytic metal ultimately governs the selectivity. In other words, the properties of the catalytic metal inherently determine the catalytic pathway and products of these plasmon-driven reactions.

**Figure 6:**
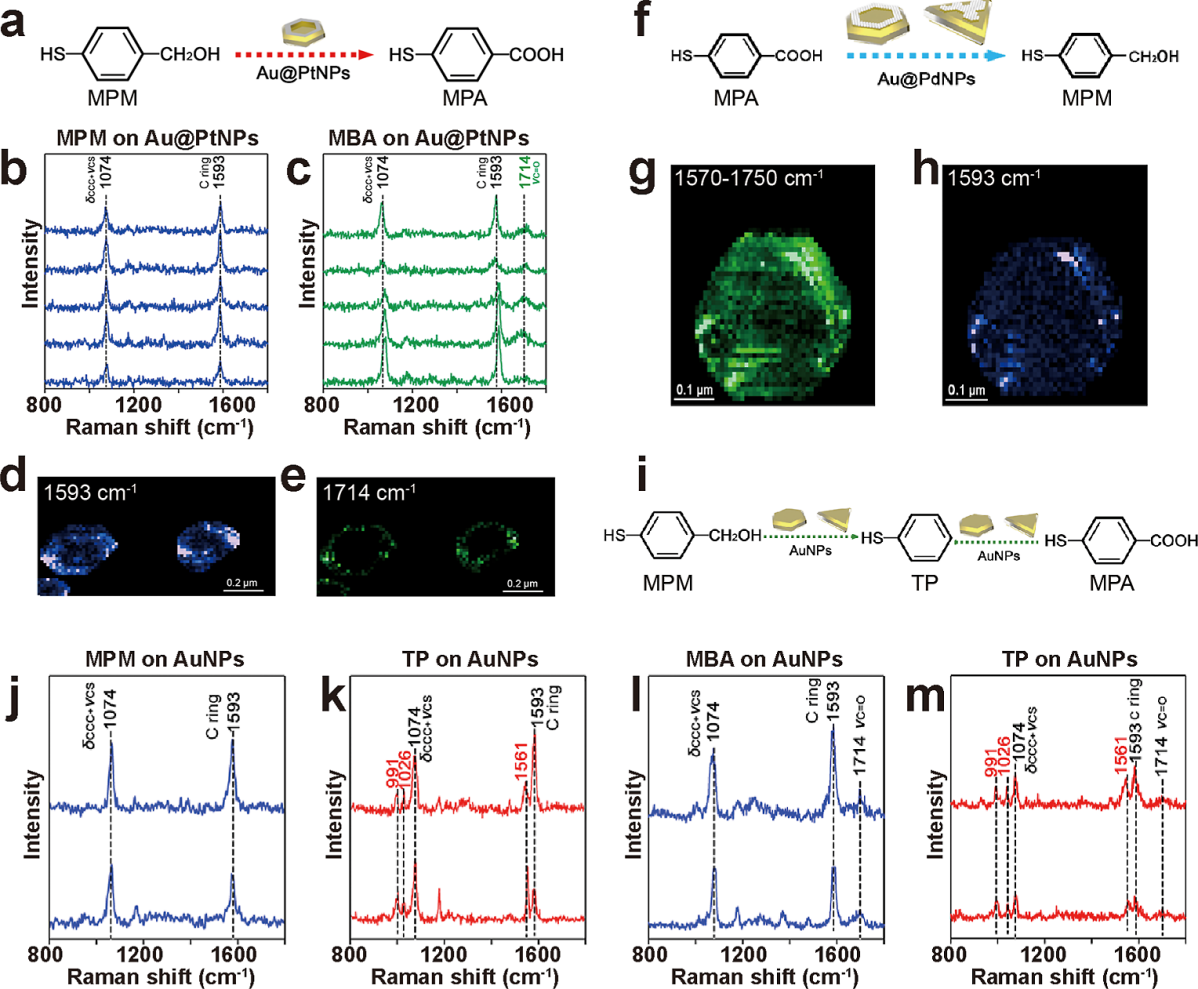
Reaction selectivity revealed by TERS mapping. (a) Schematic of the plasmon-driven oxidation of MPM to MBA on Au@PtNPs. (b,c) Typical TERS spectra extracted from the TERS maps of MPM SAM on Au@PtNPs (d,e) showing the presence of MPM (blue, b) and MBA (green, c). (d,e) TERS peak intensity maps (1593 cm^–1^, (d); 1714 cm^–1^, (e) of MPM SAM on Au@PtNPs. Step size: 10 nm. (f). Schematic of the plasmon-driven reduction of MBA to MPM on Au@PdNPs. (g,h) TERS peak intensity maps (1570–1750 cm^–1^ containing the C=C and C=O vibrations of MBA, g; 1593 cm^–1^ corresponding to the C=C vibrations of MPM, (h) of MBA SAM on Au@PdNPs. Step size: 10 nm. (i) Schematic of the C–C cleavage in MBA and MPM that leads to the formation of TP on AuNPs. (j,k) Typical TERS spectra extracted from the TERS maps of MPM SAM on AuNPs showing the presence of MPM (blue, j) and TP (red, k). (l,m) Typical TERS spectra extracted from the TERS maps of MBA SAM on AuNPs showing the presence of MBA (blue, j) and TP (red, k). Reprinted and adapted with permission from ref 94 Copyright 2021 American Chemical Society.

The same group further explored plasmon-driven catalytic selectivity using mono- and bimetallic nanostructures for the redox chemistry of *p*ATP and *p*NTP.^95,96^ Their earlier work demonstrated that on Au@PtNPs, *p*ATP underwent stepwise oxidation, namely first to *p*NTP and then to DMAB. On AuMPs, however, *p*ATP was oxidized directly to DMAB without any detectable intermediates.^95^ Conversely, Au@PdMPs catalyzed the reduction of *p*NTP to both *p*ATP and DMAB, while AuMPs produced only DMAB from *p*NTP.^96^ These results underscore how metal composition starkly dictates reaction pathways and selectivity. However, the fundamental mechanism by which catalytic metals, such as Pt and Pd, govern this selectivity requires deeper investigation. Future work should focus on the underlying factors, such as the adsorption geometry of reactants on different metal surfaces, as discussed previously, to elucidate the precise role of the catalytic metal in regulating these selective pathways.

### 2.5 Operando Studies

One of the primary goals of operando studies in heterogeneous catalysis is to establish an intrinsic correlation between the surface structure of catalysts under reaction conditions and their corresponding catalytic performance, including activity, selectivity, deactivation, and poisoning resistance. To achieve this, it is essential to simultaneously monitor the structure of catalysts and the formation of products *in situ*.^97^ As mentioned above, TERS is compatible with measurements performed in liquid under electrochemical control, known as electrochemical TERS (EC-TERS).^98–100^ EC-TERS enables the *in situ* identification of catalytically active sites at the nanometer scale during electrocatalytic reactions.

Pfisterer et al. employed EC-TERS to image the oxidation of nanoscale protrusions at a Au(111) single-crystal electrode, with a spatial resolution of ∼10 nm.^101^ These protrusions, identified as Au oxide (AuO_*x*_), were generated by electrochemical water splitting at defect sites (Figure 7a). Cyclic voltammogram (CV) of Au(111) in 0.1 M H_2_SO_4_ showed a broad shoulder peak from 1.32 to 1.48 V before the oxidation of Au terraces at 1.5 V, corresponding to the oxidation of selective oxidation of nanoscale defects. This was demonstrated by the presence of the Raman band at 560–580 cm^–1^ that was assigned to the AuO_*x*_ compounds induced by water splitting, when the potential of the sample was maintained at 1.45 V (Figure 7b). EC-TERS maps and colocalized STM images further show that the AuO_*x*_ peak at around 580 cm^–1^ is only present at the Au defect area, but not at the terraces (Figure 7c,d). By correlating the apparent height of the defect structures with the peak intensity and position of Au oxide in the EC-TERS spectra, they found that the maximum film thickness of AuO_*x*_ is approximately 3 nm. A detailed analysis of the AuO_*x*_ peak in the TER spectra acquired at various locations of the Au defect area was performed. For relatively flat areas on the defect, the peak is located above 565 cm^–1^ corresponding to the Au–O vibrations of Au_2_O_3_ (Figure 7e–f), whereas for protrusions, the Raman shift is smaller than 565 cm^–1^ corresponding to the Au–O vibrations of Au_2_O (Figure 7g). All these results contribute to the understanding of the origin of the broad and asymmetric peak shape of AuO_*x*_ in electrochemistry and demonstrate the capabilities of EC-TERS in resolving the spatial distribution of active sites in electrocatalysis. While this work provides valuable insights into the electrooxidation of Au(111), the formation and evolution of such defects are not sufficiently explored. A detailed understanding of the initial stages of defect evolution would require a higher spatial resolution, on the order of a few nanometers. Notably, such advancements have emerged. A recent study by El-Khoury demonstrated TERS imaging of chemical reactions in H_2_O with a spatial resolution of ∼3 nm.^102^ Applying this improved resolution to electrochemical systems would enable a more detailed investigation of the growth of defects at metal electrodes.

**Figure 7:**
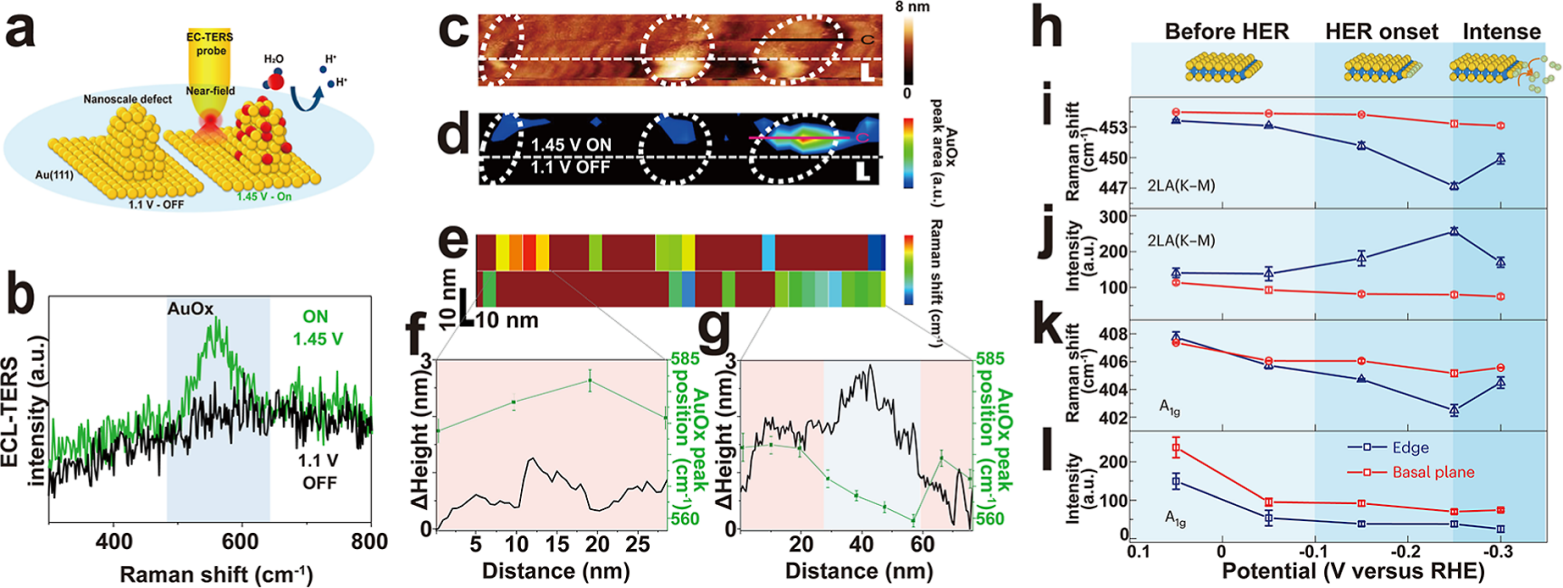
Visualization of the real–time reaction dynamics using TERS. (a) Schematic of probing the defect oxidation ON (right, 1.45 V vs Pd–H) and OFF (left, 1.1 V vs Pd–H) states of a Au(111) single crystal electrode using EC-TERS. (b) EC-TER spectra for ON (at 1.45 V) and OFF (at 1.1 V) states acquired at the defect site. (c,d) TERS intensity map of the AuO_*x*_ Raman band (d) and the corresponding STM image (c) of the Au(111) substrate. Experimental conditions: *I* = 1 nA, *E*_tip_ = 1.0 V vs Pd–H, *E*_sample_ as indicated. Scale bar: 10 nm. (e) TERS intensity map of the AuO_*x*_ Raman band extracted from the upper part of panel d. (f,g) Correlation between the EC-TERS AuO_*x*_ peak positions and Δ_height_ profiles, which are extracted from the TERS map in panel (e) and the corresponding STM image in panel (c), respectively. The error bars represent the standard deviation of the bootstrapping fitting analysis. Reprinted and adapted with permission from ref 101 Copyright 2019 Springer Nature. (h). Schematic of the hydrogen coverage at the edge of MoS_2_ in different HER stages. Yellow and green spheres represent sulfur and hydrogen atoms, respectively. (i–l) Potential dependence of the peak positions (i,k) and intensities (j,l) of the two Raman modes, namely the 2LA (K–M) mode and the A_1g_ mode, at the edge and basal plane of MoS_2_. Reprinted and adapted with permission from ref 103 Copyright 2024 Springer Nature.

Recently, significant progress has been made by the Ren group in probing the structure evolution of active sites in MoS_2_ during the hydrogen evolution reaction (HER), demonstrated by EC-TERS mapping.^103^ To gain a comprehensive understanding of the HER processes, a detailed characterization of the relationship between the surface structures and catalytic performance was conducted. When the potential was tuned from 0.05 V to −0.3 V (versus RHE) corresponding to different stages of the HER (Figure 7h), the TERS peaks of the MoS_2_ edge, in particular the A_1g_ mode (405 cm^–1^) and the 2LA (K–M) mode (450 cm^–1^) originating from the van Hove singularity in the vibrational density of states at the saddle point along the KM direction, displayed an apparent evolution for both peak position and intensity. However, both peaks in the EC-TERS spectra of the basal plane remained almost unchanged, as shown in Figure 7i–l. This direct evidence confirmed that the active site for the HER was the edge of MoS_2_, as reported in other works.^104,105^ Moreover, EC-TERS line scan was used to explore the dynamics of the active site and to visualize the synergistic evolution of atoms around it. The TERS line scan revealed an expansion of the lattice reconstruction region at the edge of a MoS_2_ bilayer during electrochemical activation, resulting from the adsorption and desorption of hydrogen atoms leading to the loss of sulfur atoms at the edge. This work provides a comprehensive characterization of the relationship between surface structures and catalytic performance, thereby revealing the intrinsic nature of MoS_2_’s active sites under operating conditions. However, key intermediates during HER, such as adsorbed interfacial water and highly reactive hydride species, were not detected in the TERS spectra. This limitation is likely due to the insufficient Raman enhancement from a single TERS hotspot for these transient species. Directly probing these intermediates under working conditions is crucial for a complete mechanistic understanding and remains a critical goal for future technical advancements in this field.

Similarly, Chen and co-workers employed the combined EC-TERS and DFT calculations to investigate the structure evolution and deactivation pathway of iron­(II) phthalocyanine (FePc) during the oxygen reduction reaction (ORR).^106^ The presence of the Raman peaks at 594, 682, and 751 cm^–1^ in the TER spectra indicated the formation of a nonplanar geometry of FePc during ORR catalysis at 0.4 V. Under extended cathodic polarization, both the appearance of the new peaks at 724 and 795 cm^–1^ and the attenuation of the peaks at 593 and 793 cm^–1^ in the TER spectra demonstrated the direct demetalation of FePc to form H_2_Pc during ORR. This is clear evidence to support the argument that the degradation mechanism of FePc during ORR involves a direct demetalation process.^106,107^

## 3 Conclusion and Outlook

In this review, recent progress in the use of TERS for the structural, temporal, and spatially resolved analysis of heterogeneous catalysts has been highlighted. With the ultrahigh spatial resolution and high chemical sensitivity, TERS has demonstrated its capability to gain valuable insights into heterogeneous catalysis, including resolving the spatial distribution of active sites in catalysis and revealing the activation pathway and chemical selectivity, even under working conditions.

Despite the significant breakthroughs achieved by TERS in heterogeneous catalysis, considerable challenges persist, particularly in extending the technique’s applicability to industrially relevant catalysts. To date, the majority of TERS investigations have focused on highly idealized systems, such as thiolate self-assembled monolayers on atomically flat gold or silver substrates. In stark contrast, industrial catalysts are complex three-dimensional composites, often comprising metal nanoparticles dispersed on high-surface-area oxide supports (e.g., Pd/CeO_2_ and Cu/CeO_2_ catalysts) and metal clusters.^108–114^ The intrinsic surface-sensitivity of TERS makes probing the chemical environment within the pores or beneath the surface layer of such materials exceptionally difficult. One promising strategy to overcome this limitation involves integrating TERS with advanced sample preparation methods, such as ultramicrotomy, to create smooth cross sections that expose the catalyst’s interior for analysis.^115^ Furthermore, the operational conditions of industrial catalysis under elevated temperatures and pressures (tens of bars for Fischer–Tropsch synthesis) pose severe challenges for TERS.^116^ These conditions can destabilize the tip–sample junction and degrade the plasmonic activity of the tip. The following discussion elaborates on these challenges and outlines potential strategies to advance TERS for broader application in heterogeneous catalysis.

### 3.1 Fabrication of Highly Active and Stable TERS Tips

Developing TERS tips with strong and stable plasmonic enhancement is paramount, as the tip is the cornerstone of the technique, dictating both spatial resolution and chemical sensitivity. Most TERS studies for the application of heterogeneous catalysis are demonstrated with the gap-mode TERS on coinage metal surfaces, such as Au and Ag. However, it is still quite difficult to probe the reactive species with the nongap-mode TERS or on noncoinage metal surfaces, such as Pt and Pd (typical catalytic metal), owing to the relatively low TERS enhancement on these surfaces in the visible light range. In addition, when the surface coverage of the reactive species is low, the detection of the TERS signal is rather challenging, even on coinage metals. This calls for the fabrication of highly active TERS tips with high reproducibility. To achieve this goal, the combination of simulations and accurate nanofabrication techniques, such as focused ion beam milling,^117,118^ electron bombardment,^119^ and field-directed sputtering,^120^ can be carried out to fabricate highly active TERS tips with optimal radii of the curvature and the geometry. Another critical challenge is maintaining this enhancement in operando conditions, where the tip must remain stable against dynamic changes at the catalyst surface. The tip instability is a primary source of poor reproducibility, a long-standing issue in the field. Therefore, future progress hinges on fabricating more robust tips and integrating them into systems engineered for exceptional vibrational and electronic stability to ensure reliable feedback control. Promising strategies to mitigate tip degradation, such as applying protective coatings to the plasmonic nanostructure, have already been demonstrated to effectively prevent the laser-induced inactivation of the tip.^121^

### 3.2 Thermal Drift

Most studies of heterogeneous catalysis using TERS are conducted at ambient or ultralow temperatures. However, many heterogeneous catalytic reactions are performed at high temperatures or high pressures. The reaction conditions at high temperatures give rise to a challenge in performing STM-TERS imaging under stable tunneling conditions to achieve high resolution images. Thus, it is desired to develop TERS equipment that could work under those relevant conditions. For STM-TERS, thermal drift mainly results from the temperature difference between a catalyst and the STM tip. This makes it difficult to achieve high resolution images at high temperatures. Fortunately, operating STM at a high temperature of 230 °C and a low pressure (30 mbar) has been demonstrated to achieve atom-resolved images.^122^ In addition, placing the tip close to the sample surface by 20–100 nm during the heating process of the catalyst before imaging could largely reduce the time to reach an approximate thermal equilibrium with the sample.^122,123^ Moreover, introducing ultrafast TERS imaging techniques can further reduce the thermal drift of the system.^124^ It can be foreseen that developing TERS equipment that can perform at high temperatures or high pressures can significantly expand the application scope of TERS in catalysis.

### 3.3 Temporal Resolution

TERS provides information on the surface species with a high spatial resolution down to the single chemical bond level.^125^ However, the temporal resolution of TERS is still limited in the time scale of seconds, which is limited by the small Raman cross-section of the surface species. It usually takes minutes to hours to obtain an eligible TERS map. However, the interfacial reactions typically occur in the picosecond to femtosecond range. Thus, it is still quite difficult to monitor the surface reaction with TERS in real time. If the temporal resolution of TERS can be further improved to the picosecond or femtosecond level, it will enable the investigation of the dynamics of surface reactions with nanoscale spatial resolution. To this end, coupling ultrafast optical systems with TERS is one way to overcome this obstacle. Some pioneering works have been done in either ambient TERS^126,127^ or UHV-TERS,^128,129^ systems, demonstrating the successful implementation of TERS with ultrashort laser pulses.^124^ Moreover, overcoming the potential reactivity of the tip itself by appropriate coating of dielectric materials is beneficial to monitor the surface reactions.^121^ As mentioned above, fabricating highly active TERS tips can also effectively reduce the recording time of the TERS signal. We believe that the development of TERS with high spatiotemporal resolution will significantly deepen the understanding of heterogeneous catalysis.

### 3.4 Combination of Other Techniques

Combining TERS with other advanced surface characteristic techniques, such as *in situ* TEM, (near ambient pressure, NAP) XPS, mass spectrometry, XAS, scanning electrochemical microscopy,^124,130,131^ and other methods, can yield more comprehensive and in-depth insights into catalytic processes. While TERS provides unparalleled nanoscale chemical information on surfaces, a complete mechanistic understanding requires correlating this data with the information on the electronic structure, morphology, and reaction kinetics of surfaces. A compelling case is its synergy with NAP-XPS. While both are surface-sensitive techniques capable of probing adsorbates and intermediates under various environments, they provide fundamentally different information. NAP-XPS is capable of quantifying the averaged electronic states and chemical composition of surfaces, as demonstrated by its use in elucidating the CO_2_ activation pathway on Cu(100),^132^ and the reduction dynamics by atomic dispersed Pt at the Cu/Cu_2_O interfaces.^133^ However, a critical limitation of NAP-XPS is its spatial resolution, which is constrained by the X-ray beam size to the micrometer scale. This inherently limits its ability to probe the nanoscale heterogeneity intrinsic to industrially relevant catalysts. While both techniques have been primarily applied to model, atomically flat systems, NAP-XPS faces even more formidable challenges than TERS in analyzing rough, high-surface-area materials under operating conditions of elevated temperature and pressure. We envision that overcoming these obstacles through technical advancements will make the combined application of TERS and NAP-XPS transformative. This multitechnique strategy, potentially extended to include *in situ* TEM, XAS, and mass spectrometry, will provide a comprehensive understanding of catalytic processes.

Besides, thanks to the development of artificial intelligence (AI) and machine learning, there have now been a few reports of applying AI or machine learning in the TERS field.^130,134^ Compared with the traditional technique, the aforementioned tools help to save the acquisition time of TERS images and to accelerate the data analysis of TERS results, which is extremely useful when dealing with a large amount of imaging data. Upon overcoming the aforementioned challenges, TERS holds immense promise for establishing precise structure–activity relationships at the single-site level. A prime example is the evolution of active sites under operating conditions. Recent work by Shi et al. showed that lattice O–O ligands boost OER activity in iron-hydroxide catalysts, as corroborated by *in situ* Raman spectroscopy, XAS, and simulations, yet the pathway for their formation remains elusive.^87^ This is precisely where TERS could offer a transformative solution, by directly probing the generation of such transient species with (sub)­nanoscale spatial resolution. The resulting profound insights are crucial for the rational design of advanced catalysts, fulfilling the ultimate objective of the field.

## Vocabulary Section

Tip-enhanced Raman spectroscopy (TERS): an analytical technique that combines scanning probe microscopy and plasmon-enhanced Raman spectroscopy, providing invaluable topographic and chemical information with nanoscale spatial resolution.
Heterogeneous catalysis: a chemical process where the catalyst exists in a different phase (typically solid) than the reactants (usually liquid or gas).
Active sites: the specific locations on the surface of a solid catalyst where the chemical reaction actually takes place.
Conversion efficiency: a performance metric that describes how effectively a catalytic process transforms reactants into the desired products.
Chemical selectivity: the ability of a catalyst to favor the formation of one desired product over other possible byproducts.
